# Effects of Dietary Yellow Mealworm (*Tenebrio Molitor*) supplementation on meat and structural egg quality of different aged-laying hens

**DOI:** 10.1016/j.psj.2025.105849

**Published:** 2025-09-15

**Authors:** Zhilong Lan, Gaixia Qiao, Xulei Ni, Qixin Yan, Ke Li, Minjie Zhang, Xinyi Liu, Liu Liu, Chune Zhang, Xinbao Liu, Ying Luo

**Affiliations:** a College of Food Engineering and Nutritional Science, Shaanxi Normal University, Xian, Shaanxi, 710119, China; b Ningxia Hui Autonomous Region Grain and Oil Product Quality Inspection Center, Yinchuan 750001, China; c State Key Laboratory of Efficient Production of Forest Resources, Yinchuan 750004, China

**Keywords:** Tenebrio molitor, Insect meal, Laying hen, Meat quality, Age-specific response

## Abstract

Global poultry production faces dual pressures of sustainable protein scarcity and age-specific nutritional demands. Tenebrio molitor (**TM**) emerges as an eco-friendly alternative protein source, yet its differential effects across laying hen life stages remain unquantified. This study aimed to identify optimal TM dietary inclusion levels for pre-laying (96-day-old) and peak-laying (236-day-old) hens by evaluating key performance indicators, including meat quality, nutritive indices, antioxidant enzyme activities, and structural egg quality. Eighty uniform-weight Hy-Line Brown hens (60-day-old and 200-day-old) were randomly assigned by age to four dietary groups (0 %, 5 %, 10 %, or 20 % TM) for a 35-day period, with ten replicates per group. Results demonstrated age-dependent responses to TM supplementation. In 96-day-old hens, most TM levels were ineffective. Conversely, in 236-day-old hens, 10 % TM addition level optimally improved meat texture, while maintaining antioxidant capacity, and enhanced eggshell strength. Based on these findings, we propose an age-stratified TM supplementation strategy: less than 5 % supplementation for pre-laying hens and 10 % for peak-laying hens. This precision nutrition approach enhances insect protein utilization efficiency in poultry farming while optimizing the eating quality and nutritional value of poultry products from laying hens.

## Introduction

Amid escalating global concerns regarding climate change and ecological preservation, the poultry industry faces mounting pressure to reconcile production demands with sustainable resource utilization (Oke et al., 2024). The growing demand for sustainable and nutrient-dense feed ingredients in poultry production has driven research into alternative protein sources. Among these, Tenebrio Molitor (**TM**) larvae can efficiently utilize agricultural by-products and convert them into high-quality biomass proteins, reducing feed production costs (Derler et al., 2021; Morales-Ramos et al., 2020). Nowadays, TM has emerged as a promising candidate due to its rich nutritional composition and favorable environmental sustainability profile. TM larvae are characterized by high protein content, approximately 50-70 % of dry matter, a balanced amino acid profile (though sometimes limited in sulfur-containing amino acids), and significant levels of unsaturated fatty acids and minerals such as calcium (Ca), manganese (Mn), and zinc (Zn) (Dragojlović et al., 2022; Liceaga, 2022; Shafique et al., 2021; van der Heide et al., 2021). What’s more, TM is also rich in bioactive compounds such as functional polysaccharides and antimicrobial peptides. These components not only provide essential nutrients but may also enhance immune function and gut health in animals, positioning TM as a multifunctional feed additive (Syahrulawal et al., 2023).

Studies on dietary TM supplementation have demonstrated positive outcomes across various species. In aquaculture, dietary supplementation with TM has been demonstrated to yield beneficial effects in several fish species, including Ctenopharyngodon idellus (Li et al., 2023), Oncorhynchus mykiss (Jeong et al., 2020) and Monopterus albus (Yuan et al., 2024). The addition of TM to the diet has been shown to significantly enhance antioxidant capacity, immune activity and survival rates in these species. Furthermore, Zhang and others found that replacing fish meal with 15 % defatted TM meal can improve the Juvenile Large Yellow Croakers's immunity to a certain extent, improve the intestinal structure and microbial composition, and finally enhance intestinal health (J. Zhang et al., 2022). Beyond aquaculture, TM supplementation has also demonstrated efficacy in livestock diets. In compared with fish meal, meat meal or meat-bone meal, TM addition can significantly improve the digestibility of dry matter, crude protein, total amino acids, essential amino acids and non-essential amino acids of pigs, it suggesting broad applicability of TM in livestock nutrition (Yoo et al., 2019). Additional, replacing TM up to 10.0 % in the Japanese quail diet can promoted weight gain, improved feed conversion, and significantly increase carcass weight, meat quality, and the content of some amino acids and saturated fatty acids. These findings underscore the potential of TM to serve as a viable alternative to conventional protein sources like soybean meal and fishmeal, which face sustainability challenges.

In poultry, preliminary research highlights the safety of TM supplementation and its potential benefits on growth performance, dietary quality and egg production, 4 % dry TM supplement can significantly increase the body weight and daily gain of broilers during brooding and improve feed conversion (Elahi et al., 2020). In another study, Dabbou found that dietary substitution of corn gluten meal with 7.5 % TM significantly increased the percentages of oleic acid (C18:1 c9) and α-linolenic acid (C18:3 n-3) in breast muscle of Label Hubbard hybrid free-range chickens (Dabbou et al., 2020). Furthermore, TM supplementation can also enhance significantly eggshell structure parameters, including shell thickness and break strength, while concomitantly reducing both hen body mass and egg weight (Ait-Kaki et al., 2022).

However, existing studies predominantly focus on single growth stages, leaving a critical gap in understanding how the nutritional and physiological responses to mealworm supplementation vary across different phases of a hen’s lifecycle. Therefore, this study investigated the effects of TM supplementation on Hy-line Brown laying hens at two key developmental stages: pre-laying (60-96 days) and peak-laying (200-236 days). The present research aimed to determine the differences in the effects of TM on different-aged laying hens by evaluating meat quality, nutritive indexes, and structural egg quality. These findings will advance precision nutrition strategies in poultry husbandry, providing mechanistic insights to optimize TM inclusion protocols according to hens' developmental requirements, thereby enhancing both economic viability and production sustainability.

## Material and methods

### Material

60-day-old and 200-day-old Hy-Line Brown laying hens were randomly selected from local farms, ensuring health and uniformity in body weight and condition. Total protein assay kits, along with T-AOC, CAT, SOD, and GSH-Px detection kits, were procured from the Nanjing Jiancheng Bioengineering Institute (Nanjing, China). All other chemicals were commercially obtained from domestic suppliers and were of analytical grade, with solutions prepared using deionized water. All animal experiments were conducted in accordance with the Guidelines for the Care and Use of Experimental Animals approved by the State Council of the People’s Republic of China. The study protocol was reviewed and approved by the Academic Committee and the Office of Science and Technology at Shaanxi Normal University.

### Experimental design and laying hens management

A total of 80 Hy-line Brown laying hens with uniform body weight were selected, with ages of 60 days and 200 days, respectively. Each age group was randomly divided into 4 treatment groups, with 10 replicates per group. The hens were fed either a basal diet (Table 1) or experimental diets supplemented with 5 %, 10 %, and 20 % TM in the basal diet, respectively, over a period of 35 days. The hens were housed in stacked cages (150 cm × 60 cm × 70 cm) within a greenhouse maintaind at 24-28°C. A light regimen of 16L:8D (light: dark) cycle was applied. During the initial 7 days, the hens were fed a standard diet. Subsequently, they were fed according to treatment regimens three times daily at 7:30, 14:00 and 18:00. The basal diet was formulated according to modified NRC (1994) and Chinese Chicken Feeding Standard (NY/T 33-2004) guidelines. Eggs were collected daily during the final three days of the experiment. Three eggs per group were randomly selected for analysis, resulting in a total of 36 eggs examined. On day 36 of the experiment, three hens per group were randomly selected and euthanized by cervical dislocation, resulting in a total of 24 hens. Breast muscle samples were collected and divided into two aliquots: one was stored at 4°C for short-term analysis, and the other was stored at −80°C for long-term preservation and subsequent analysis.

**Table 1:** The ingredients of the basal diet and nutritional levels used in the experiment.

Items	Amount
Ingredients (%)	
Corn	60
Soybean meal	25
Limestone	8
Soybean oil	2
Premix^1^	5
Total	100
Nutrient levels	
Metabolizable energy, MJ/kg	11.62
Crude protein, %	17.49
Calcium, %	3.94
Total phosphorus, %	0.58
Analyzed composition CP, %	0.38
Methionine, %	0.46
Lysine, %	1.04

1 The premix provided per kilogram of compound in the diet: Vitamin A, 14,000 IU; vitamin D_3_, 7, 000 IU; vitamin E, 71 IU; vitamin B_1_, 2.625 mg; vitamin B_2_, 10.5 mg; vitamin B_6_, 5.25 mg; vitamin B_12_, 0.02 mg; vitamin C, 80 mg; folic acid, 1.05 mg; biotin, 0.07 mg; niacin, 35 mg; pantothenic acid, 15.75 mg; Fe, 80 mg; Cu, 8 mg; Mn, 100 mg; Zn 60 mg; Co, 0.2 mg; I, 0.5 mg; Se, 0.4 mg; choline chloride (50 %), 1.05 g; l-Lys, 1.36 g; l-Met, 1.71 g; NaHCO_3_, 1 g; NaCl, 3 g; mildew-proof agent, 4 g.

### Meat quality detection

pH measurement. pH values were measured within 4 h post-slaughter. Breast muscle samples (1.0 g) were weighed and homogenized at a 1:9 (w/v) ratio with normal saline at 12,000 r/min for 1 min. Then the homogenate was measured using a SevenCompact™ pH meter (Mettler Toledo S210, Switzerland).

Drip loss (**DL**) determination. DL was assessed as described by Wang et al. (2023). 100 g of fresh breast muscle (m_1_, g) were hung with a suspended vertically in sealed polyethylene bags with sterile hooks at 4°C for 24 h. After carefully blotting surface exudate with filter paper, the samples were reweighed (m₂, g). DL (%) is calculated as equation: DL=[(m_1_-m_2_)/m_1_]×100 %

Texture Profile Analysis (**TPA**) measurement. According to the method described by Ali et al. (2023), breast muscle samples were sectioned into uniform cubes (1 cm × 1 cm × 1 cm) parallel to the muscle fiber orientation. The textural properties were evaluated using a texture analyzer (TA. XT. Plus, Stable Micro Systems, UK) equipped with a cylindrical compression probe (P/36R, contact surface area = 1017.88 mm²). The testing protocol conducted at a constant crosshead speed of 1 mm/s with 50 % strain deformation. The fundamental texture parameters hardness (N), gumminess (N), chewiness (mJ), springiness (mm), cohesiveness (Ratio), and resilience (%) were recorded.

Microstructure observation. Scanning Electron Microscopy (**SEM**) analysis was carried out to evaluate how different treatment methods affected the chicken breast samples. Cubic samples sized 2 × 2 × 2 (mm) were fixed in 2.5 % glutaraldehyde at 4°C overnight. Subsequently, samples were dehydrated through a graded ethanol series and freeze-dried for 24 h. Finally, samples were observed using SEM (Nova NanoSEM 450, FEI Company, Hillsboro, USA) at 800× magnification.

### Nutritive indexes detection

Total protein (**TP**) measurement. A 1.0 g breast muscle sample was homogenized with PBS to prepare a 0.5 % (w/v) solution using ultrasonication. The mixture was centrifuged at 4,000 r/min for 10 min, and the supernatant was collected. TP was quantified using commercial kits (Jiancheng Bioengineering, Nanjing, China) following the manufacturer's instructions.

Amino acid composition measurement. A 0.1 g breast muscle sample was accurately weighed and hydrolyzed in 10 mL of 6 mol/L hydrochloric acid at 110°C for 18 h. The hydrolysate was evaporated to dryness under reduced pressure, then reconstituted with 0.1 mol/L HCl to a final volume of 25 mL. The solution was further diluted 500-fold to prepare the test sample. Pre-column derivatization was performed using phenyl isothiocyanate (PITC), followed by HPLC analysis with a binary mobile phase: solvent A (0.1 mol/L sodium acetate buffer-acetonitrile, 93:7 v/v) and solvent B (acetonitrile-water, 4:1 v/v). Operating conditions were: flow rate 1.0 mL/min, column temperature 40°C, detection wavelength 254 nm, and injection volume 5 μL.

### Antioxidant enzyme activities measurement

The breast muscle samples were mixed with PBS at a 1:9 (w/v) ratio, then centrifuged at 4,000 r/min for 10 min. The supernatant was collected to determine activities of total antioxidant capacity (**T-AOC**), glutathione peroxidase (**GSH-Px**), superoxide dismutase (**SOD**), and catalase (**CAT**) using commercial kits (Jiancheng Bioengineering, Nanjing, China) following the manufacturer's protocol.

### Structural egg quality detection of 236 days aged laying hens

Eggshell strength measurement. The break strength was measured using a texture analyzer (TA.XT.Plus, Stable Micro Systems, UK) equipped with a cylindrical compression probe (P/36R, contact surface area = 1017.88 mm²). The protocol was conducted at 1 mm/s crosshead speed with 5 % strain deformation.

Egg shape index (**ESI**) measurement. The transverse diameter (l_1,_ mm) and longitudinal diameter (l_2_, mm) of the egg were measured using vernier caliper, The ESI (dimensionless) is calculated as equation: ESI = l_2_/l_1_

### Statistical analyses

All data were processed using Microsoft Excel 2019, and analyzed by one-way ANOVA using GraphPad Prism 10.1.2. For significant differences (*p* < 0.05), Tukey's HSD test was used for multiple comparisons. Graphs were plotted using GraphPad Prism 10.1.2. Results are presented as mean ± standard error (SE).

## Results and discussion

### The effect of TM supplementation on meat quality of different-aged laying hens

**Fig. 1:**
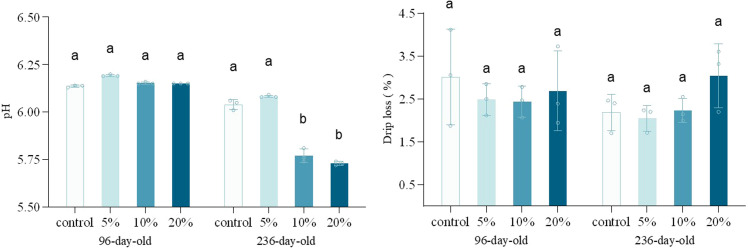
pH and drip loss of different-aged laying hens with different concentration of TM addition. Different litters indicate significant differences compared to the control groups (*p* < 0.05).

Fig. 1 displays the pH and drip loss of different-aged laying hens. In 96-day-old hens, pH values remained basically constant under all supplementation levels without statistical differences. In contrast, 236-day-old hens showed a threshold effect, pH values decreased with 5 % supplementation groups maintaining pH 6.1, while 10 % and 20 % supplementation groups exhibited lower pH at pH 5.8 and 5.7. The results regarding pH values demonstrated significant concordance with the results reported by Selaledi and others (2021), wherein high-level TM supplementation induced significantly lower postmortem ultimate pH values in poultry meat compared to conventional diets. Anaerobic glycolysis serves as the predominant metabolic pathway in postmortem muscle tissue, driving lactate accumulation and consequently inducing a rapid pH decline (Beauclercq et al., 2022; Zhang et al., 2023). Adult chickens exhibit higher trypsin activity compared to chicks, and the TM is a high-protein feed addition, thereby excess amino acids may be converted to glycogen via the gluconeogenic pathway, ultimately accelerating postmortem glycolysis and subsequent pH decline in breast muscle (Millar and Harding, 2019).

Water-holding capacity (**WHC**) is the ability of post-mortem muscle tissue to retain its inherent water under external force. Water loss during cooking directly affects the juiciness and tenderness of meat. (Nikbakhtzade et al., 2024), and the drip loss is a significant indicate of WHC. Although the pH values in 10 % and 20 % supplementation groups were significant, the influence of TM supplementation on the drip loss was not statistically significant in either the 96-day-old hens or the 236-day-old hens. In the study conducted by Murawska et al. (2021), comparable findings were observed wherein the substitution of soybean meal with Full-Fat Black Soldier Fly *(Hermetia illucens L.*) Larvae Meal in broiler diets did not exert significant effects on drip loss rate of poultry meat. Generally speaking, the drip loss rate of meat is negatively correlated with its pH value (Jankowiak et al., 2021; Tarczyński et al., 2021). But meanwhile, the high-protein diet may enhance WHC by promoting the growth of muscle fiber, thereby effectively counteracting the adverse effects induced by pH decline (D. Zhang et al., 2024).

### The effects of TM supplementaion on TPA of different-aged laying hens

**Fig. 2:**
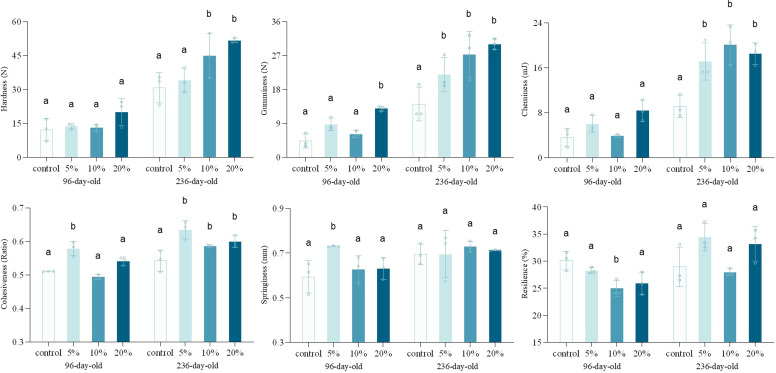
Hardness, gumminess, cheminess, cohesiveness, springiness and resilience of different-aged laying hens with different concentration of TM addition. Different litters indicate significant differences compared to the control groups (*p* < 0.05).

TPA is considered one of the most critical mechanical testing methods for determining consumer acceptance of food products. It employs a reciprocating motion to compress the meat sample twice, simulating the mandibular movement during mastication (Alfaifi et al., 2023). The effects of TM supplementation on TPA with hardness, gumminess, chewiness, cohesiveness, springiness, and resilience were shown in Fig. 2. The results showed that TM supplementation levels on hardness, gumminess, chewiness exhibited age-dependent divergence. In 96-day-old hens, the 20 % supplementation group showed a modest increase in hardness and chewiness, but no significant difference was observed. In gumminess, a dose-dependent upward trend was observed as well, but only the 20 % supplementation group reached a value of 12.88 N, showed significance. Cohesiveness seemingly followed quadratic trends, the 5 % supplementation group showed a significant increase, maintaining a value of 0.579, whereas 10 % and 20 % supplementation groups were no significant differences. In 236-day-old hens, hardness, gumminess and chewiness positively correlated with TM supplementation, both 10 % and 20 % supplementation groups significantly exceeded the control group. Additionally, the effect on gumminess and chewiness were more significant, 5 % supplementation group exhibited significantly higher than control group. Cohesiveness peaked at 5 % supplementation level, with maintained at 0.635, which was significantly higher than control group, and two higher-dose groups higher significantly as well. The effect of springiness and resilience between different-aged hens were not significant. In 96-day-old hens, only the 5 % supplementation group showed significantly higher springiness (0.730 mm), while 10 % and 20 % supplementation group did not differ. In addition, the 10 % supplementation group demonstrated significantly lower resilience of 24.97 % than the control group, while the 5 % and 20 % groups showed no statistical differences, no significant difference in springiness or resilience were observed across all supplementation levels in 236-day-old hens. Knowing that 100 % of muscle fibers in chicken breast muscle are Type IIB fibers, the observed differences in meat hardness may primarily arise from variations in muscle fiber diameter (Wang et al., 2024; Weng et al., 2022). Studies have demonstrated a negative correlation between muscle fiber diameter and meat tenderness, with smaller fiber diameters and higher fiber densities contributing to a finer meat texture and higher hardness values corresponded to prolonged mastication duration (Huo et al., 2021). Thus, the increased values of hardness, chewiness, and gumminess in hens may be attributed to the increase of muscle fiber diameter. Meanwhile, the values of hardness, chewiness, and gumminess may be enhanced by the presence of myofibrillar proteins in the meat matrix, these proteins enhance compressive resistance, thereby facilitating the formation of a denser internal network structure (Kamani et al., 2019).

**Fig. 3:**
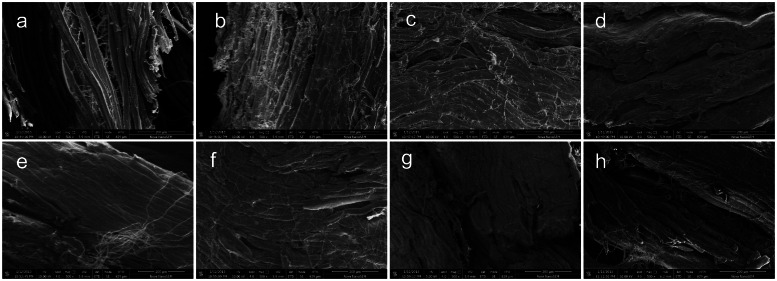
SEM image of different-aged laying hens with TM addition. a-d were 96-day-old laying hens with 0, 5 %, 10 %, and 20 % of TM addition, respectively; e-h were 236-day-old laying hens with 0, 5 %, 10 %, and 20 % of TM addition, respectively.

Muscle fibers can be classified into two categories based on their contractile velocity: slow-twitch (Type I) fibers and fast-twitch (Type II) fibers (Matarneh et al., 2021). Type IIB fibers exhibit a larger diameter with significantly greater cross-sectional area and volume compared to other muscle fiber types (HOSOTANI et al., 2021). The growth characteristics of muscle fibers were shown in Fig. 3. In the 236-day-old hens, the diameter of muscle fibers in the high-dose group was significantly large. In general, the length and cross-sectional area of muscle fibers exhibit progressive increases with advancing age, while dietary regimens significantly modulate this developmental process (Ali et al., 2021). In brief, high-protein and high-fat diets in poultry may lead to an increased proportion of Type IIB fibers (fast-glycolytic fibers) within the skeletal muscle tissue (D. Zhang et al., 2024). It has been substantiated that Type IIB fibers exhibit accelerated postmortem glycolytic rates, typically manifesting lower ultimate pH values and concomitantly higher textural hardness, findings that are consistent with the experimental outcomes of the present study (Cheng et al., 2021; LeMaster et al., 2023).

Fig. 4

**Fig. 4:**
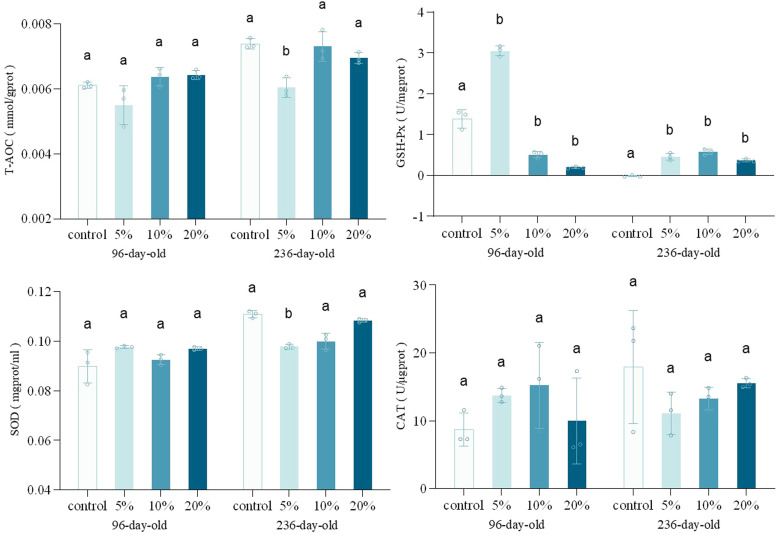
T-AOC, GSH-Px, SOD, and CAT of different-aged laying hens with different concentration of TM addition. Different litters indicate significant differences compared to the control groups (*p* < 0.05).

### The effect of TM supplementaion on meat nutritional quality of different-aged laying hens

TP levels in laying hens at different ages were presented in Table 2. No significant difference was observed in the 96-day-old hens. Conversely, in the 236-day-old hens, a quadratic relationship was observed between the protein content of broiler meat and the supplementation level of TM, the 5 % supplementation group demonstrated a TP content of 190 g/kg in breast muscle, showing statistically significant increased compared to the control group, whereas the 10 % and 20 % supplementation groups showed no statistically significant differences. However, the present result was not quite consistent with that another report showing a quadratic correlation between TM supplementation levels and TP content, with the TP reaching its lowest point at a 4 % supplementation level (Elahi et al., 2020), maybe the discrepancy potentially attributable to divergent processing protocols of TM meal. It has been proved that the mTOR signaling pathway stimulates intramuscular protein synthesis within myofibers in avian muscle development (Xu and Velleman, 2023). However, high-protein diets may decrease key proteolytic enzyme activities (particularly pepsin and trypsin) while inducing intestinal inflammation. These combined effects ultimately suppress mTOR-mediated anabolic signaling, thereby reducing protein content in the muscle (Chen et al., 2021; Zhang et al., 2020).

**Table 2:** Breast muscle amino acid composition (μmol/g) and TP (mg/g) of different-aged laying hens with different concentration of TM addition. Different litters indicate significant differences compared to the control groups (*p* < 0.05).

**Item**	96-day-old groups				236-day-old groups			
	Control	5 %	10 %	20 %	Control	5 %	10 %	20 %
TP	201.64±7.75	188.71±7.75	192.69±9.85	189.04±5.41	169.47±6.62^a^	190.7 ± 11.17^b^	185.72±9.15^a^	177.1 ± 5.71^a^
EAA								
Val	97.46±0.06^a^	99.01±0.08^b^	97.85±0.09^b^	101.51±1.06^a^	88.29±0.24^a^	93.88±0.08^b^	98.07±0.08^b^	99.16±0.09^b^
Met	51.11±0.01^a^	50.54±0.06^b^	48.15±0.05^b^	50.87±0.03^b^	45.62±0.04^a^	47.64±0.02^b^	51.14±0.03^b^	53.89±0.06^b^
Ile	97.68±0.09^a^	98.97±0.07^b^	94.54±0.02^b^	100.47±0.07^b^	87.86±0.27^a^	93.59±0.11^b^	99.6 ± 0.16^b^	103.75±0.16^b^
Leu	165.67±0.1^a^	166.89±0.27^b^	161.78±0.21^b^	170.67±0.1^b^	147.31±0.04^a^	157.15±0.13^b^	168.6 ± 0.13^b^	175.45±0.06^b^
Phe	60.74±0.02^a^	61.3 ± 0.09^b^	59.99±0.04^b^	62.76±0.04^b^	54.57±0.09^a^	58.07±0.08^b^	61.77±0.02^b^	63.45±0.03^b^
Lys	137.28±0.06^a^	139.48±0.12^b^	134.68±0.39^b^	148.48±0.29^b^	126.78±0.61^a^	135.99±0.29^b^	146.89±0.07^b^	149.95±0.08^b^
His	59.11±0.05^a^	62.17±0.1^b^	61.54±0.24^b^	67.84±0.01^b^	63.79±0.09^a^	66.26±0.19^b^	70.22±0.01^b^	67.41±0.06^b^
ΣEAA	669.06±0.39^a^	678.36±0.81^b^	658.53±1.05^b^	702.59±1.6^b^	614.22±1.38^a^	652.6 ± 0.91^b^	696.29±0.49^b^	713.07±0.53^b^
NEAA								
Glu	241.35±1.71^a^	232.27±13.66^a^	240.28±5.43^a^	187.57±4.72^b^	253.72±30.81	198.89±2.97	227.64±12.73	264.56±0.45
Ala	88.69±1.07	84.38±1.96	103.39±8.23	75.09±9.79	124.28±11.61	100.11±6.63	93.93±2.29	82.25±1.05
Cys	82.74±0.58	81.5 ± 0.47	84.22±1.46	75.48±5.85	69.64±2.51^a^	75.11±1.27^a^	78.44±0.41^a^	84.13±0.86^b^
Tyr	46.46±0.05^a^	46.82±0.04^b^	45.34±0.03^b^	47.33±0.15^b^	41.25±0.06^a^	44.37±0.17^b^	47.01±0.05^b^	49.68±0.11^b^
Arg	106.02±0.07^a^	105.7 ± 0.09^a^	106.17±0.76^a^	107.27±0.06^b^	92.8 ± 0.17^a^	99.49±0.56^b^	107.21±0.21^b^	110.85±0.11^b^
ΣNEAA	565.27±3.47^a^	550.67±16.23^a^	579.4 ± 15.91^a^	492.73±20.57^b^	581.69±45.15^a^	517.97±11.6^b^	554.24±15.69^a^	591.47±2.57^a^
ΣAA	1234.32±3.86^a^	1229.03±17.03^a^	1237.93±16.96^a^	1195.32±22.16^b^	1195.91±46.54^a^	1170.57±12.55^a^	1250.53±16.18^b^	1304.54±3.1^b^

EAA: essential amino acid; ΣEAA: sum of essential amino acids; NEAA: non-essential amino acid; ΣNEAA: sum of non-essential amino acids; ΣAA: sum of total amino acids.

As fundamental units of proteins, adequate and proportionally balanced amino acids promote health, growth, and reproduction in chickens, simultaneously, muscle amino acid profiles critically govern meat flavor and nutritional value (S. Liu et al., 2023). Table 2 presented the effects of TM supplementation levels on hens at different ages. In the 96-day-old hens, all TM levels significantly increased essential amino acid content, peaking at 702.59 μmol/g with 20 % TM. However, 20 % supplementation caused a significant glutamate reduction, ultimately decreasing total amino acids. In the 236-day-old hens, TM more markedly enhanced EAAs: 13 % and 16 % increases with 10 % and 20 % supplementation, respectively. Concurrently, non-essential amino acids showed no significant reduction, thereby significantly increasing total amino acid content. Glutamate is essential for rapid growth in poultry, participating in nitrogen balance and energy supply (He et al., 2021), 96-day-old hens are still in the growth phase with rapid gonadal development (Lu et al., 2023). Therefore, TM-induced glutamate reduction may impair growth rate, potentially delaying the onset of egg-laying. In 236-day-old hens, total amino acids exhibited greater variation than total protein content, potentially attributed to free amino acids in muscle. However, TM supplementation significantly increased essential amino acid levels, thereby enhancing nutritional quality.

### The effect of TM supplementaion on antioxidant activity of different-aged laying hens

**Fig. 5:**
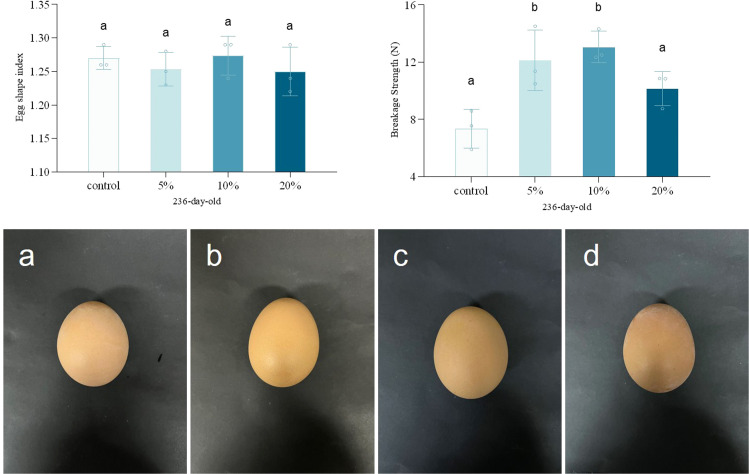
Egg shape index, eggshell strength and image of different-aged laying hens with different concentration of TM addition. a-d were 236-day-old laying hens with 0, 5 %, 10 %, and 20 % of TM addition, respectively. Different litters indicate significant differences compared to the control groups (*p* < 0.05).

T-AOC is commonly employed to assess antioxidant potential, whereas GSH-Px, SOD, and CAT constitute critical components of redox homeostasis and the antioxidant defense system (Wei et al., 2019; Yao et al., 2023). The levels of T-AOC, GSH-Px, SOD, and CAT in laying hens at different ages were presented in Fig. 5. In 96-day-old hens, no significant differences were detected in antioxidant indices except for GSH-Px across supplementation levels, while antioxidant parameters exhibited a quadratic relationship with supplementation levels in 236-day-old hens. Specifically, the 5 % supplementation group demonstrated significantly lower T-AOC and SOD activity, whereas the 10 % and 20 % supplementation groups showed no statistically significant differences. Mitochondria serve as primary sources of antioxidant enzymes. Compared to slow-twitch (Type I) fibers, fast-twitch (Type II) fibers exhibited reduced antioxidant enzyme activities, including T-AOC and SOD, due to their reduced mitochondrial density (Hosotani et al., 2021; Liu et al., 2022). Chicken meat is rich in polyunsaturated fatty acids and is prone to lipid oxidation, resulting in off-odors, surface discoloration, protein denaturation, and nutrient loss, which reduces consumer acceptability. The endogenous antioxidant system in meat, comprising antioxidant enzymes, peptides, and proteins, acts as metal ion chelators or free radical scavengers to maintain meat quality stability and extend shelf life (Xiong et al., 2020). Consequently, a 5 % TM supplementation level may result in decreased meat preservation characteristics in laying hens.

### The effect of TM supple mentaion on structural egg quality of 236-day-old laying hens

The eggshell is a composite material consisting of a calcite mineral phase and an organic phase. Its shape and strength determines the breakage rate during production and transportation. Therefore, eggshell shape and strength is critical to global food security, given the egg’s role as a staple food (Fu et al., 2024; Gautron et al., 2021). For laying hens, research on the ESI holds multifaceted core value, encompassing hatchability, genetic breeding programs, optimization of egg quality, production management, and economic benefits (Gutiérrez et al., 2021; Đermanović et al., 2024). As shown in Fig. 6, all the ESI of supplementation groups (5 %, 10 %, 20 %) showed values ranging from 0.77 to 0.81, with no statistically significant differences. Fig. 6 demonstrates a quadratic relationship between TM supplementation level and eggshell strength, peaking at 10 % (13.0 N). Zn serves as an essential cofactor for carbonic anhydrase, which supplies the necessary carbonate precursors for the formation of calcium carbonate (CaCO₃)—the main component of the eggshell. Meanwhile, Mn acts as a key activator for glycosyltransferases, enzymes that catalyze the synthesis of glycosaminoglycans (GAGs) and proteoglycans, directly influencing the initiation of mineralization and the regulation of crystal morphology (Palanisamy et al., 2023; Y. Zhang et al., 2022). As a feed rich in both Zn and Mn, TM supplementation thereby enhances eggshell strength, a result consistent with the findings of Ait-Kaki et al. (2022). However, the decline beyond 10 % TM requires further investigation.

## Conclusion

To address the critical gap in age-specific responses to sustainable protein supplementation, this study investigated the effects of dietary TM supplementation on Hy-line Brown laying hens at two developmental stages, through comprehensive assessments of meat quality and egg quality. The present study demonstrated that the 10 % TM supplementation level was optimal for 236-day-old hens, primarily promoting egg and meat quality. Regarding meat quality, the 10 % TM supplementation significantly improved texture parameters, enhancing suitability for processing. Crucially, the antioxidant capacity remained stable, avoiding the decline observed at lower (5 %) TM levels. For egg quality, it significantly increased eggshell strength to a peak compared to controls. However, potential drawbacks include a reduced breast meat pH in 236-day-old hens, potentially accelerating post-mortem glycolysis and increasing drip loss, necessitating rapid chilling. For 96-day-old hens, TM supplementation did not significantly alter most meat quality, total protein content and antioxidant enzyme activities. The sole exception was increase in cohesiveness and gumminess at the 5 % TM supplementation level. Collectively, for pre-laying hens, not more than 5 % TM supplementation is recommended to avoid cost-ineffectiveness, whereas 10 % supplementation is advised for peak-laying hens to enhance meat quality and eggshell strength. Our findings empower precision nutrition strategies that align insect protein supplementation with hen physiological maturity, advancing both economic and environmental sustainability in the poultry industry, while optimizing eating quality and nutritional value of laying hens.

## CRediT authorship contribution statement

**Zhilong Lan:** Writing – review & editing, Writing – original draft, Visualization, Validation, Project administration, Methodology, Investigation, Formal analysis, Data curation. **Gaixia Qiao:** Resources, Project administration, Methodology, Investigation, Funding acquisition, Conceptualization. **Xulei Ni:** Writing – review & editing, Methodology, Investigation, Formal analysis, Data curation. **Qixin Yan:** Writing – review & editing, Validation, Investigation. **Ke Li:** Validation, Resources, Investigation. **Minjie Zhang:** Resources, Investigation. **Xinyi Liu:** Resources, Investigation. **Liu Liu:** Resources, Funding acquisition. **Chune Zhang:** Resources, Funding acquisition. **Xinbao Liu:** Resources, Funding acquisition. **Ying Luo:** Writing – review & editing, Supervision, Project administration, Methodology, Funding acquisition, Conceptualization.

## Disclosures

The authors declare that they have no known competing financial interests or personal relationships that could have appeared to influence the work reported in this paper.
